# Stability of Propolis Phenolics during Ultrasound-Assisted Extraction Procedures

**DOI:** 10.3390/foods13132020

**Published:** 2024-06-26

**Authors:** Mladenka Malenica, Magdalena Biesaga, Sandra Pedisić, Lara Saftić Martinović

**Affiliations:** 1 Department of Medical Chemistry, Biochemistry and Clinical Chemistry, Faculty of Medicine, University of Rijeka, 51000 Rijeka, Croatia; mladenka.malenica@medri.uniri.hr; 2 Faculty of Chemistry, University of Warsaw, 1 Pasteur Str., 02-093 Warsaw, Poland; mbiesaga@chem.uw.edu.pl; 3 Faculty of Food Technology and Biotechnology, University of Zagreb, 10000 Zagreb, Croatia; spedisic@pbf.hr; 4 Department of Medical Biology and Genetics, Faculty of Medicine, University of Rijeka, 51000 Rijeka, Croatia; 5 Department of Biotechnology, University of Rijeka, 51000 Rijeka, Croatia

**Keywords:** heat reflux extraction, mass spectrometry, phenolic acids, flavonoids, phenolic recovery, complex matrix

## Abstract

Propolis has gained popularity in recent years as a potential preventive and therapeutic agent due to its numerous health benefits, which include immune system boosting, blood pressure lowering, allergy treatment, and skin disease treatment. The pharmacological activity of propolis is primarily attributed to phenolics and their interactions with other compounds. Given that phenols account for most of propolis’s biological activity, various extraction methods are being developed. The resin–wax composition of the propolis matrix necessitates the development of an extraction procedure capable of breaking matrix–phenol bonds while maintaining phenol stability. Therefore, the aim of this study was to assess the stability of two major groups of phenolic compounds, flavonoids and phenolic acids, in propolis methanol/water 50/50 (*v*/*v*) extracts obtained after ultrasound-assisted extraction (USE) under different extraction parameters (extraction time and pH) and heat reflux extraction (HRE). The methodology involved varying the USE parameters, including extraction time (5, 10, and 15 min) and pH (2 and 7), followed by analysis using liquid chromatography–tandem mass spectrometry (LC-MS/MS) to quantify phenolic recoveries. Results revealed that benzoic acid and chlorogenic acid derivatives demonstrated excellent stability across all ultrasound extraction procedures. The recoveries of flavonoids were highly diverse, with luteolin, quercitrin, and hesperetin being the most stable. Overall, neutral pH improved flavonoid recovery, whereas phenolic acids remained more stable at pH = 2. The most important optimization parameter was USE time, and it was discovered that 15 min of ultrasound resulted in the best recoveries for most of the phenols tested, implying that phenols bind strongly to the propolis matrix and require ultrasound to break the bond. However, the high variability in phenol extraction and recovery after spiking the propolis sample shows that no single extraction method can produce the highest yield of all phenols tested. As a result, when working with a complex matrix like propolis, the extraction techniques and procedures for each phenol need to be optimized.

## 1. Introduction

Propolis is a natural and complex resinous material made by honeybees to produce the necessary “glue” that will cement their cracked hives. Its complexity is reflected through its high-diversity composition: wax, resins, balsams, pollen, essential and aromatic oils, and other organic substances [1]. Due to its composition, propolis acts as a solid material in cold conditions, but it turns to soft and sticky gum at higher temperatures.

Although the main propolis components remain the same regardless of the propolis type, bioactive constituents can greatly vary depending on their geographical origin [2]. The constituents that show the highest diversity and add value to this natural product are phenols—antioxidant compounds responsible for their antibacterial, antifungal, antiviral, and anti-inflammatory activity [3].

As a procedure for polyphenol isolation, ethanol extraction is most commonly used, due to the simplicity of maceration as an extraction procedure, ethanol accessibility, and the low cost [4]. The maceration process is time-consuming and significantly prolongs the time needed for the extract preparation. Therefore, extraction procedures with other solvents such as methanol, isopropanol, and chloroform, sometimes with the addition of glycerol, polyethylene glycol, and many different oils, along with pH value adjustments, were developed [5,6]. Although methanol was shown to be successful in polyphenol extraction, the addition of water in the final solvent is desirable because water causes swelling of the sample, and therefore increases the sample surface, which allows better solvent penetration and higher polyphenol yield. As a result, in this paper, methanol and water were used as solvents for polyphenol extraction. To shorten the extraction time, the maceration process is often assisted with microwave (MAE) or ultrasound (USE) extractions. Methods based on these techniques reduce the amount of the needed solvent and simplify the extraction process. In comparison, it was shown that USE is more selective and efficient because it causes high diffusion rates across the sample content, which allows better solvent penetration into the matrix, but at the same time without the extraction of non-phenolic compounds, as can be seen when using MAE [5]. This extraction technology uses high-frequency sound waves to generate cavitation bubbles in a liquid medium. Cavitation’s mechanical effects disrupt cell walls, increasing solvent penetration and making intracellular compounds more easily released. This results in higher yields of desired extracts in a shorter period than traditional methods. In addition, USE uses low temperatures, which is critical for maintaining polyphenol integrity and bioactivity. High temperatures in traditional extraction methods can degrade sensitive compounds, reducing antioxidant capacity and overall quality. In terms of phenol extraction, USE technology works with a variety of solvents, including water, ethanol, and other organic solvents. This adaptability enables the optimization of extraction conditions tailored to specific polyphenol types and applications, thereby increasing the overall efficiency and selectivity of the extraction process. Still, USE is an invasive method because of the poor stability of some phenols, mainly those with the highest antioxidant activity, i.e., flavonoids. Therefore, USE parameters such as frequency, power, time, and wave distribution should be optimized according to the analyzed sample.

Despite many studies about total polyphenol yield after different extraction procedures assisted with ultrasonic waves, there is a lack of information about the recovery of specific flavonoids and phenolic acids. Few reports on phenol recovery and stability were conducted on natural sources such as grape seeds [7], apples [8], rosemary, sage [9], honey [10], etc., but there are no reports on phenol recovery after different extraction procedures derived from propolis samples. Regardless of some known information about polyphenol content of propolis or other natural sources which bear great similarity to propolis, Biesaga and Pyrzynska [11] showed that even small differences in matrix composition strongly influence the success rate of extraction and the stability of phenolic compounds, which means that results obtained on other sources potentially cannot apply to propolis.

The main challenge in propolis extraction is extracting phenols from the very complex structure while maintaining the stability of flavonoids and phenolic acids. Owing to the high content of resin and wax in propolis, the diversity of structures and chemical properties of phenolic acids and flavonoids, the first step in optimizing the extraction procedure is to choose the appropriate solvent and USE time to achieve good isolation of phenols from the matrix, but at the same time to avoid the formation of free radicals and complex polyphenol forms. Therefore, in this paper, we addressed six scientific problems: (1) complexity of propolis matrix, (2) optimization of USE parameters, (3) influence of solvent composition and changes in pH, (4) stability of phenolic compounds, (5) lack of data on propolis extraction, and (6) challenges in development of analytical method for its determination.

The primary goal of this study is to assess the stability and recovery of flavonoids and phenolic acids in propolis extracts via ultrasound-assisted extraction (USE). The study’s goal is to optimize extraction parameters such as solvent composition and ultrasonic duration in order to maximize polyphenol yield while preserving the compounds’ integrity and biological activity. The study focuses on the efficacy of a methanol/water (50/50 *v*/*v*) solvent system at various pH levels (pH = 2 and pH = 7) and ultrasonic durations (5, 10, and 15 min). The extracted phenolic compounds will be examined using liquid chromatography–tandem mass spectrometry (LC-MS/MS) to determine their recovery rates and stability.

## 2. Materials and Methods

### 2.1. Samples

The propolis sample was obtained from Croatia in 2016 and stored in a dark and cold place before use.

### 2.2. Chemicals

Sigma (Steinheim, Germany) supplied the commercial phenolic standards: gallic acid, 3,4-DHBA, chlorogenic acid, *p*HBA, vanillic acid, *p*-coumaric acid, quercitrin, luteolin, quercetin, naringenin, apigenin, kaempferol, hesperetin, rhamnetin, and pinocembrin. Merck (Darmstadt, Germany) supplied HPLC gradient-grade methanol and acetonitrile. All experiments used ultra-pure water from the Milli-Q system (Millipore, Bedford, MA, USA), which has an electrical resistivity of 18 Qm.

### 2.3. Extraction Procedures

Prior to the extraction, the propolis sample was triturated in a mortar and then used for different extraction modes. For each extraction mode, the sample, standards, and sample spiked with 1 ppm of flavonoid and phenolic acid standards or with 1 ppm of mixture of all phenol standards were analyzed in two repetitions. After the extraction step, the sample, standardl and spiked sample were filtrated thought a PTFE membrane filter of 0.45 µm and then injected into the LC-MS/MS instrument for analysis. The total concentration of each phenol was converted to mg/g. The recovery of each phenolic component was determined by subtracting the concentration of the unspiked sample from the spiked sample. Since 1 ppm of each phenolic component was initially added to the soldered sample, it was determined how much of that 1 ppm remained after the extraction procedure. The results are shown in the form of percentage.

### 2.4. Ultrasonic Extraction (USE)

A total of 0.0034 g of propolis was weighed and mixed with methanol/water (50/50 *v*/*v*) solvent to make a 2 mL final solution volume. The obtained solution was spiked with 1 ppm of phenolic standard mixture. The prepared sample was placed in a glass vial (6 × 1 cm) and immersed in an ultrasonic bath (Polsonic, Warsaw, Poland). Ultrasound characteristics were as follows: 2 × 100 W ultrasonic power (peak/period), frequency 540 kHz, and power of heating 150 W. During the ultrasound, the water bath temperature was kept below 30 °C. The first analysis included different USE times of 5, 10, and 15 min, as well as pH values of 2 and 7. All analyses were conducted in duplicate (N = 2). The mean values of the analyzed and spiked propolis samples after the USE procedure were used to calculate recovery. The obtained percentage is the average of two measurements.

### 2.5. Heated Reflux Extraction (HRE)

HRE is a solid–liquid extraction method that preserves solvent by repeating solvent evaporation and condensation at a set temperature for a predetermined amount of time. This extraction process was used as a control to USE extraction.

A total of 0.0034 g of propolis was weighed and mixed with methanol/water (50/50 *v*/*v*) solvent to make a 2 mL final solution volume. The pH of the solution was adjusted to 2 using formic acid. The obtained solution was spiked with 1 ppm of the phenolic standard mixture. The prepared sample was placed in a glass vial (6 × 1 cm) and then heated in a boiling water bath (95 °C) for 15 min. All analyses were conducted in duplicate (N = 2). The mean values of the analyzed and spiked propolis samples after the HRE procedure were used to calculate recovery. The obtained percentage is the average of two measurements.

### 2.6. LC-MS/MS Conditions

Chromatographic analysis was carried out using a Shimadzu LC system that included binary pumps LC20-AD, a degasser DGU-20A5, a column oven CTO-20AC, and an autosampler SIL-20AC (Shimadzu, Kyoto, Japan), all of which were connected to a 3200 QTRAP Mass spectrometer (Applied Biosystem (Foster City, CA, USA)/MDS SCIEX) via an additional Valco valve. An MS system with an electrospray ionization source (ESI) was run in negative-ion mode. The ESI conditions were as follows: capillary temperature 450 °C, curtain gas at 0.3 MPa, auxiliary gas at 0.3 MPa, negative ionization mode source voltage −4.5 kV, and positive mode 4.5 kV. Nitrogen was used as both a curtain and an auxiliary gas. The optimal Multiple Reaction Mode (MRM) conditions for each compound were determined in the infusion mode (Table 1). Standard solutions were infused into the electrospray source via a 50 μm i.d. PEEK capillary using a Harward Apparatus pump at 10 μL per minute. Continuous mass spectra were obtained by scanning *m*/*z* between 50 and 650.

Compounds were separated using a Luna C-18 (2) (Phenomenex) column (100 × 2.1 mm, 3 µm) with precolumn at 30 °C. Eluents A and B were 8 mM formic acid (pH 2.8) and acetonitrile, respectively. The mobile phase was delivered at a rate of 0.2 mL/min in gradient mode as follows: 0–3 min linear gradient from 1% to 10%B, 3–10 min from 10% to 20%B, 10–15 min from 20% to 25%B, 15–20 min from 25% to 30%B, 20–25 min 30%B, 25–30 min from 30% to 90%B, 30–31 90%B, 31–32 from 90% to 10%B. Post time was set to 18 min. The flow rate was 0.2 mL/min.

The analytes were identified by comparing the retention time and *m*/*z* values obtained by MS and MS2 to the mass spectra of standards tested under identical conditions. Detailed method development and optimization were previously published in a paper by Biesaga and K. Pyrzynska [12]. All analyses were conducted in duplicate (N = 2).

### 2.7. Statistical Analysis

The statistical analysis of the data obtained from the propolis extraction experiments was performed using Microsoft Excel (Microsoft 365 version 2405 (Microsoft)).

## 3. Results and Discussion

**Figure 1:**
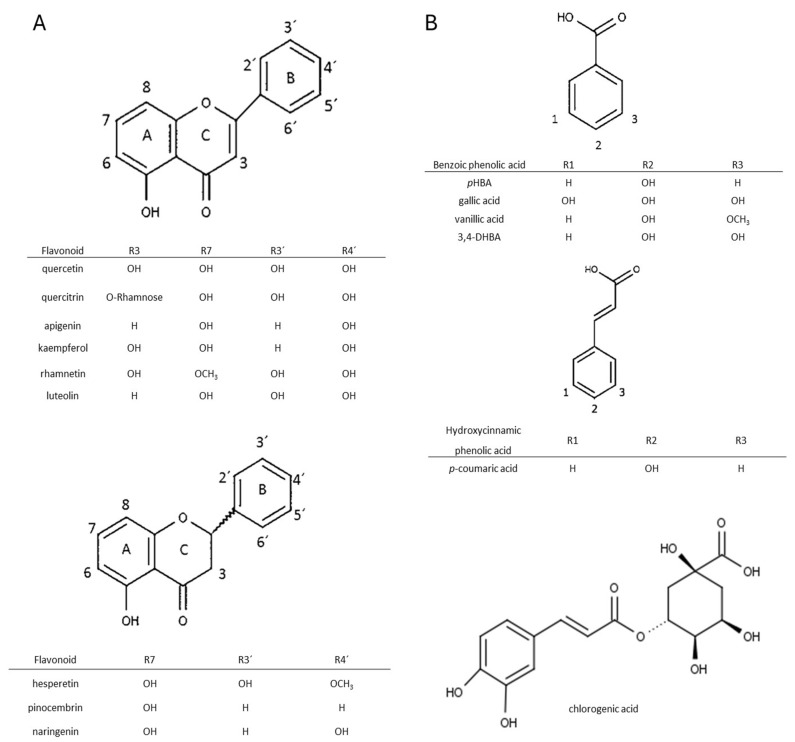
Chemical structures of the studied flavonoids (**A**) and phenolic acids (**B**). Basic structures were prepared in Chemsketch (Version 2021).

Flavonoids and phenolic acids are two major types of phenolic compounds. Figure 1 shows their respective structures. Herein, an explanation of research methodology and the experiments that were conducted prior to the analysis of the recovery of phenolic components from propolis are shown. The recovery of phenolic acids and flavonoids will be covered in more detail in the subsections.

The work plan was to investigate the ultrasonic extraction behavior of pure phenolic components and when extracted from propolis. Heat reflux extraction (HRE) served as a control. In the first step, a mixture of all analyzed phenols was prepared, and their stability in methanol solution was determined after a 5, 10, and 15 min USE.

**Figure 2:**
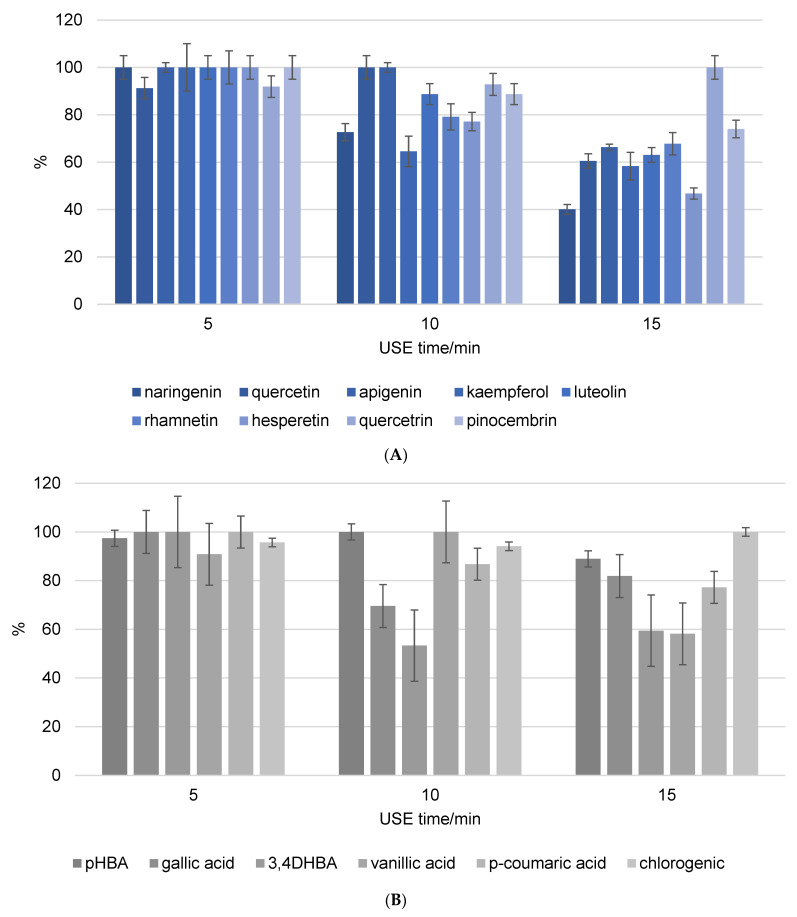
Recovery of flavonoid (**A**) and phenolic acid (**B**) standard mixture exposure to different ultrasound time (5, 10, and 15 min). Results were recalculated as the highest stability value at 100% of the recovery.

Figure 2 depicts the stability of flavonoids and phenolic acids in methanol solution, respectively. The objective was to compare the stability and recovery rates of flavonoids and phenolic acids when subjected to USE for different durations (5, 10, and 15 min). The recovery rates of the phenolic compounds were measured and recalculated, setting the highest stability value observed as 100%. This normalization allows for a straightforward comparison of how the duration of ultrasound affects the stability and recovery of each compound in methanol. The figures clearly show that the phenolic components in the methanol solution decompose as USE time increases. Naringenin and hesperetin were among the most decomposed flavonoids, with decomposition rates exceeding 50% after 15 min of USE. The unsaturation of the C ring in combination with the 4-oxo group on the C ring significantly increases the biological activity of the compounds. As a result, it is possible to conclude that components with a high biological content decompose more quickly, as evidenced by the findings in this study. In addition, quercetin is a very strong antioxidant and consequently an unstable phenolic compound, while quercitrin is its glycosylated form. Glycosylated flavonoids have greater stability and weaker antioxidant activity than non-glycosylated forms, and the results are in line with expectations. The most decomposed phenolic acids were 3,4-DHBA and vanillic acid after 15 min of USE, with decomposition rate above 40%. The varying stability of different phenolic acids under ultrasound treatment indicates that their chemical structures have a significant influence on their resistance to degradation. The hydroxyl groups in 3,4-DHBA and vanillic acid can oxidize, particularly under the stress conditions caused by ultrasound. The methoxy group in vanillic acid may cause steric hindrance, leaving the hydroxyl group more exposed and reactive under ultrasound conditions. This can result in faster degradation than other phenolic acids without such substituents. Gallic acid was decomposed with the rate around 20% after 15 min of USE. In comparison, the additional hydroxyl group in gallic acid can have a stabilizing effect because it influences the electronic distribution within the molecule, making it less reactive to the harsh conditions produced by ultrasound cavitation.

**Figure 3:**
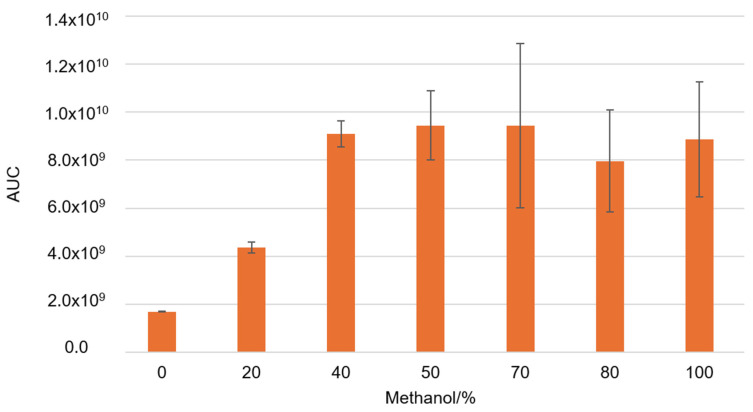
The total concentration of all analyzed phenolic components area under the curve (AUC) during ultrasonic extraction with various methanol–water ratios.

Next, we measured the concentrations of specific phenolic components in the propolis sample. A proportion of 50/50 *v*/*v* methanol and water showed the best results in total phenol extraction, so this ratio was used for further extractions (Figure 3). As a changing variable, we used different USE times and pH values of the obtained solution. This was done because phenolic components have different physicochemical properties at low and neutral pH.

To relate the physicochemical properties of analyzed phenols with our results, we utilized the logS and logD values provided in Table 2. The logD value represents the distribution coefficient, indicating the ratio of concentrations of all forms (ionized and non-ionized) of a specific phenol in a mixture of two phases: polar and nonpolar. This value allows us to calculate the molecule’s lipophilicity at different pH levels. The logS value, on the other hand, represents the molecule’s solubility at various pH levels. From Table 2, which lists the logD and logS values at pH 2 and pH 7, we observe that as the pH increases, the logD values of phenols decrease, indicating a reduction in their lipophilicity.

Therefore, an increase in pH will increase the affinity of the phenolic component to the more polar environment. This implies that the higher the pH for phenols will result in weaker lipophilic matrix–flavonoid bond and improved extraction efficiency. Additionally, higher logS values indicate better solubility in aqueous media, with values above −2 denoting soluble compounds, between −2 and −4 indicating partially soluble compounds, and below −4 signifying insoluble compounds.

The table further shows that changes in pH have a more pronounced effect on phenolic acids compared to flavonoids, as evidenced by the greater variation in their logD and logS values between neutral and acidic pH levels. This suggests that phenolic acids are more sensitive to pH changes, which significantly influence their solubility and distribution between polar and nonpolar phases. As pH rises from 2 to 7, logD values for phenolic acids decrease, indicating decreased lipophilicity. Because of their increased affinity for the polar aqueous environment, phenolic acids appear to be more soluble and easier to extract in a 50/50 methanol–water solution at higher pH levels. In terms of chemical structure, at a pH of 2 (the pH investigated in our study), all the analyzed phenolic compounds exist primarily in their protonated forms and prefer a non-polar environment, such as the propolis matrix. Similarly, flavonoids have lower logD values at higher pH, indicating that they will become more soluble in the methanol–water mixture. This will make it easier to extract the flavonoids from the propolis matrix because the polar solvent system can better penetrate and release them.

However, there is more complexity behind pH changes in propolis extraction, since the extraction solution’s low pH can disrupt the propolis matrix by affecting the wax and resin composition.

Table 3 and Table 4 show the total concentrations of analyzed phenolic components in methanol/water (50/50 *v*/*v*) propolis solutions following USE extraction (5, 10, and 15 min) at various pH levels and after HRE extraction.

**Figure 4:**
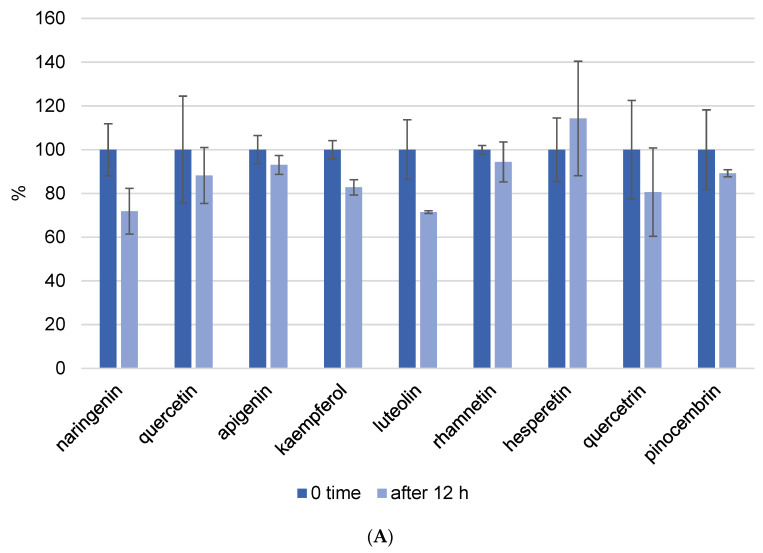

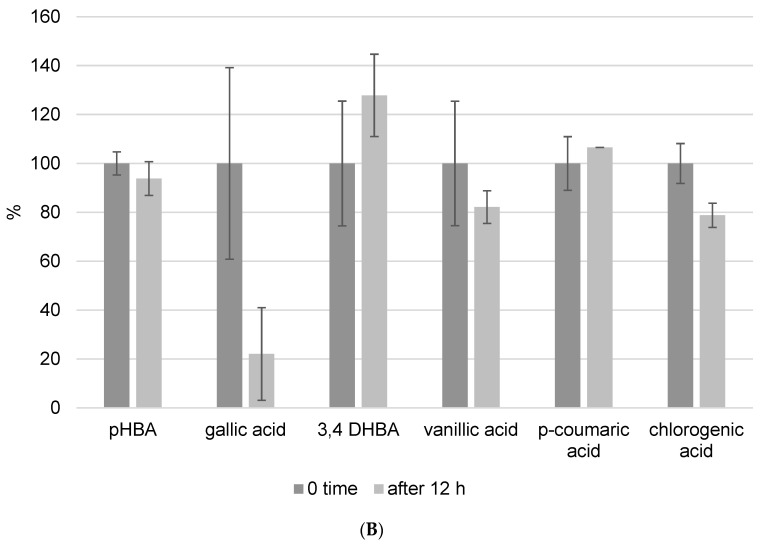
Kinetics of flavonoid (**A**) and phenolic acid (**B**) decomposition in propolis methanol/water extract after 12 h. Results were recalculated as the results in 0 min as 100% of the recovery.

Because one run of the LC-MS/MS method for one sample takes 50 min and the phenolic components are unstable, we also investigated the kinetics of decomposition by analyzing the prepared extract every hour for a period of 12 h. In this experiment, we prepared propolis extract using the following parameters: pH = 7 and a USE time of 10 min. Figure 4 depicts the kinetics of phenolic component decomposition. The graph shows the results at zero minutes and 12 h, and it is clear how certain phenols decompose with time. All analyzed flavonoids showed good stability after 12 h at room temperature (Figure 4A). However, phenolic acids did not show the same stability. Gallic acid was the most decomposed of the phenolic acids, and after 12 h, only 22% of the gallic acid remained in the methanol/water solution. This analysis was performed to determine whether the standing time of the prepared extracts in the instrument affects the result, and these findings were also considered when the analysis procedure was implemented.

After analyzing all these parameters, the propolis methanol/water extract was spiked with 1 ppm of a selected mixture of phenol standard solutions, and the recoveries were measured. The following two sections provide detailed explanations of the findings. This step was taken to investigate the behavior of phenolic components when added to a complex matrix, such as propolis. Based on their chemical nature, we assumed that flavonoids would bind more strongly to the lipophilic matrix of propolis, resulting in variable recovery values. Flavonoids are more difficult to isolate from the matrix because of their stronger binding affinity to the lipophilic components of propolis, and once isolated, they are more susceptible to degradation, particularly when subjected to prolonged ultrasound exposure. This increased susceptibility to degradation complicates their recovery and stability during the extraction procedure. As polar molecules, phenolic acids were expected to bind less tightly to the lipophilic matrix and be easier to isolate. Since we previously demonstrated that phenolic acids do not degrade after 15 min in a methanol solution, we do not anticipate degradation here, but rather high recovery.

### 3.1. Flavonoid Recoveries

The flavonoids studied in this paper were from three major polyphenol groups: flavanols, flavanones, and flavones. These compounds differ from each other according to the number and position of hydroxyl groups. Most of them contain hydroxyl groups at positions 5 and 7 in the A ring, but the main differences are among substituents and double bonds in the B and C rings (Figure 1A). In some cases, a methoxy group or attached sugar can significantly alter their physical–chemical properties, thereby influencing their pharmacological activity [14]. Most flavonoids occur naturally as glycosides with attached hexoses or pentoses. Therefore, it is necessary to achieve a good extraction procedure to break these O- or C-glycoside bonds and release flavonoids.

**Figure 5:**
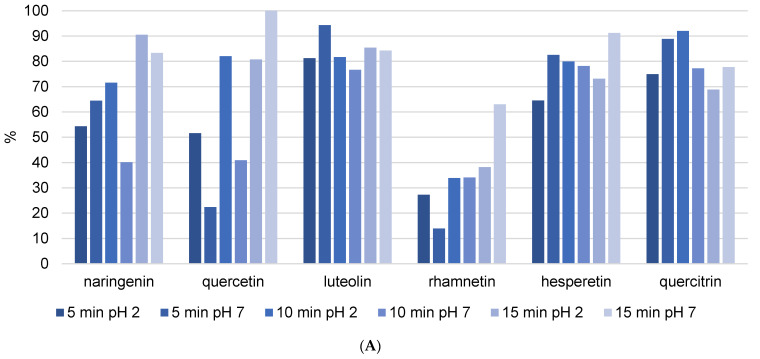

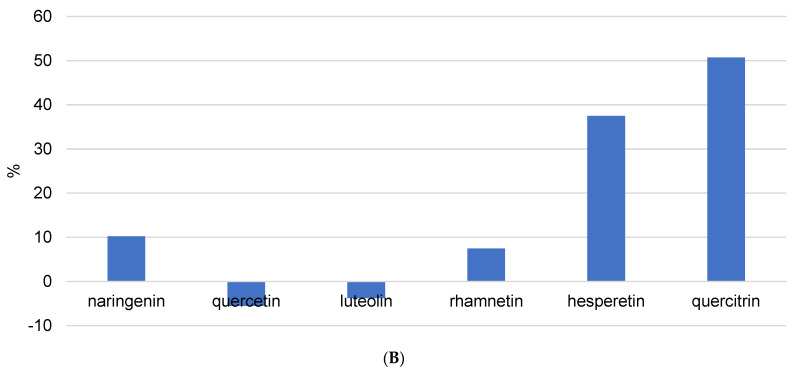
The recovery of flavonoids after (**A**) USE extraction (5, 10, and 15 min) with methanol/water (50/50 *v*/*v*) solution at different pH (2 and 7) and (**B**) HRE extraction procedure.

Analyzed flavanols quercetin, kaempferol, and rhamnetin showed high diversity among recoveries after extraction enhanced with different USE time and different pH (Figure 5A and Figure S1). These differences can be the result of different positions of hydroxyl groups. Namely, it is possible for kaempferol to have a strong connection to the resin–wax constitution, which can influence extraction success and the uniqueness of the results. Quercetin and rhamnetin showed lower recoveries, especially at 5 min and pH = 7. This can be due to its hydroxyl and methoxy (in the case of rhamnetin) substituents at positions 3, 7, 3′, and 4′. This high number of substituents can cause a steric effect and therefore lower stability of the flavonoid structure. Quercetin displayed lower recovery compared to its glycoside form quercitrin, suggesting that the presence of sugar increases flavonoid stability. It was previously demonstrated that glycosylation on position 3 in the C ring can lower their ability to release hydrogen, which lowers their pharmacological activity [15]. Recovery of the flavanone hesperetin was similar in all extraction procedures (Figure 5A). This stability can be due to the absence of the 2–3 double bond in conjugation with the 4-oxo group. It was shown that polyphenols with these features have lower antioxidant activity and therefore higher stability, so our results agree with literature [14]. Naringenin, despite belonging to the same flavonoid family as hesperetin, demonstrated an unusually different recovery. In fact, recovery increased with increasing duration of USE, potentially meaning that naringenin is more incorporated into the resin–wax composition of propolis and a longer duration of ultrasound is needed for isolation. However, the trend differs between ultrasound durations of 5 min, 10 min, and 15 min. It can be seen in Figure 5A that a higher pH with 5 min of ultrasound improves the isolation of phenolic components, whereas a longer duration of ultrasound results in better extraction at lower pH values. When we compare these results to the values from Table 2, we can see that they are completely opposite. Table 3 demonstrates that the best extraction occurred after 5 min of USE at acidic pH. Although these extraction conditions caused 50% of naringenin to decompose (Figure 5A), they also produced the highest amount of this flavonoid (Table 3). The explanation is that naringenin is a strong antioxidant; it previously demonstrated the potential for degradation in the kinetics analysis, and it is expected that shorter ultrasound durations will be more effective for its isolation.

In the case of flavones, luteolin recovery was not influenced by USE time. These results were confirmed by Table 3, where it can be seen that all extraction procedures yielded similar concentrations of luteolin. Apigenin, pinocembrin, and kaempferol are not mentioned because their recovery values were unusual (Figure S1) and could not be attributed to the physicochemical properties of each component. Table 3 shows that propolis contains very high concentrations of all three components. Pinocembrin was found in the highest concentration. Using USE = 5 min and pH = 7, 3.6332 ± 1.2276 mg/g was extracted. On the other hand, as shown in Figure S1, pinocembrin recovery is negative in some cases, and there is no trend in terms of ultrasound duration or solvent pH change. The only explanation is that it is difficult to unify the extraction procedure because propolis is a very complex matrix and USE consistently deviated from previous values. Considering these speculations, it would be necessary to analyze the reproducibility of USE as a method of isolating phenolic compounds from propolis and to repeat the same procedure dozens of times and determine the concentrations.

Heat reflux extraction is a solid–liquid extraction method that preserves solvent by repeating solvent evaporation and condensation at a set of high temperatures for a predetermined amount of time. Figure 5B and Figure S2 demonstrate the decomposition of flavonoids under high temperature in the HRE procedure. The lowest recovery rates were observed for naringenin, quercetin, luteolin, and rhamnetin, which were lower than 10%. Furthermore, all analyzed flavonoids had recoveries of less than 60%. This could be attributed to dimer (from two radicals) or quinone formation. Only quercitrin showed thermal stability (recovery above 50%) because it was protected by its glycoside form. When comparing the HRE and USE techniques (Figure 5A,B), it is clear that USE is the preferred method for flavonoid isolation; however, the USE time and pH of the extraction solution should be adjusted for each isolated compound.

### 3.2. Phenolic Acid Recoveries

Phenolic acids are classified into two types based on their structure: benzoic and cinnamic acids. Their antioxidant activity is based on their ability to form stable phenoxy radicals. These radicals are the result of the low energy of hydroxyl substituents (repercussion of the aromatic ring’s resonance effect). Specifically, a decrease in electron density in the oxygen atom causes the hydrogen atom to detach from the hydroxyl group. These radicals can either form quinones or dimerize [16].

**Figure 6:**
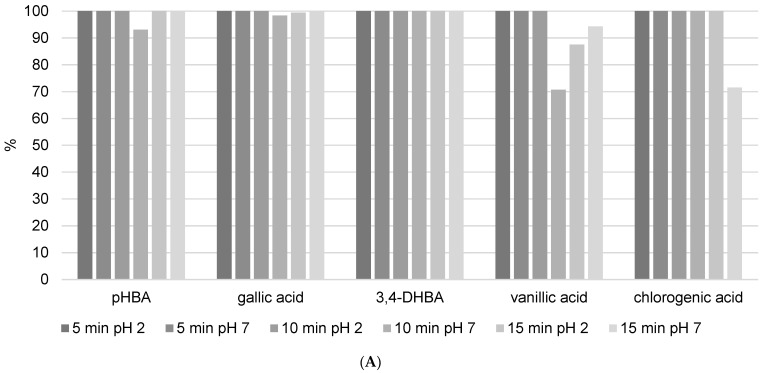

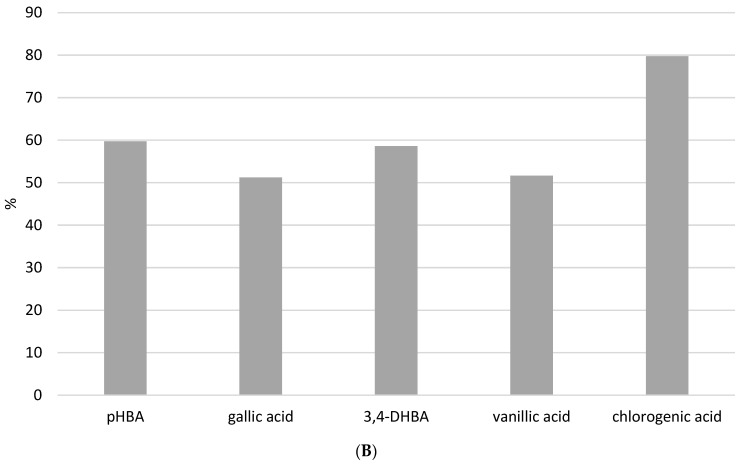
The recovery of phenolic acids after (**A**) USE extraction (5, 10, and 15 min) with methanol/water (50/50 *v*/*v*) solution at different pH (2 and 7) and (**B**) HRE extraction procedure.

Analyzed benzoic acids: 4-hydroxybenzoic acid (*p*HBA), gallic acid, and 3,4-dihydroxybenzoic acid (3,4-DHBA) showed high stability in general (Figure 6A). This is the result of the position of the carboxyl group, which has a negative influence on the release of the hydrogen ions in benzoic derivatives. Therefore, benzoic acids cannot easily discharge hydrogen ions and form stable radicals, so they have lower antioxidant activity than derivatives of cinnamic phenolic acids, and consequently are stable and have high recovery values [17]. Furthermore, differences are also observed among the same phenolic acid group. Namely, antioxidant activity strongly depends on the number of hydroxylic substituents and the steric effect in their structures [16]. It was shown that benzoic acid derivatives with one hydroxyl substituent at position 2 have low antioxidant activity and therefore are more stable [17]. This fact is confirmed in this research. *p*HBA showed high stability at different extraction modes with variable USE times. Considering the obtained results, the recommendations for *p*HBA extraction would be to use lower USE time and pH = 7. Addition of a methoxy substituent into the monohydroxy ring increases the antioxidative potential of phenolic acid. Vanillic acid has a methoxy group at position 3 (Figure 1B), and its stability is violated due to the steric effect between two close substituents. Decomposition of vanillic acid was observed at high USE time, 15 min. These results are confirmed in Table 4, where it is shown that a USE time of 5 min at pH = 2 is most suitable for vanillic acid extraction (0.1171 ± 0.0286 mg/g). Among all benzoic acid derivatives, ones with two hydroxyl substituents show higher antioxidant stability, specifically if these substituents are located at positions 1 and 3 [17]. 3,4-DHBA showed stability in all extraction modes presented in Figure 6A. Furthermore, gallic acid, which has three hydroxyl substituents, should have the lowest stability, but in this case, it showed good recovery (although the kinetics analysis of pure standard in methanol solution after 12 h in Figure 4 showed a high level of decomposition). Chlorogenic acid is a cinnamic phenolic acid derivative (Figure 1B), but also showed high recovery in analyzed extraction procedures (Figure 6A). However, Table 4 shows highly variable total concentrations. In addition, the kinetics of the reaction showed a certain degree of decomposition after 12 h. Chlorogenic acid proved to be more stable than another derivative of cinnamic acid, *p*-coumaric acid (Figure S3), which is probably due to the complex structure where unstable cinnamic base is stabilized. However, significant variations in the total amount of the extracted component demonstrate that benzoic acid derivatives remain more stable than cinnamic acid derivatives.

The recovery values and total quantity of *p*-coumaric acid (Figure S3 and Table 4), a representative of hydroxycinnamic acids, were found to be extremely variable. The answer to such behavior is that it is found in high concentrations in propolis (8.0368 ± 0.4338 mg/g with 10 min of USE and pH = 7) and causes the same problem as apigenin, pinocembrin, and kaempferol. This is logical because stronger antioxidants become radicals more easily by neutralizing free radicals and transferring themselves to other constituents. In terms of LC-MS/MS analysis, they are no longer visible as the original components, i.e., they decompose.

The standard method of HRE (Figure 6B and Figure S4) showed that heating can have a negative influence on phenolic acid stability. Recovery of all analyzed phenolic acids after the HRE procedure was below 60%, except for chlorogenic, with a recovery of around 80%. Figure 6A clearly shows that all analyzed phenolic acids remain stable under USE, so this procedure would be the preferred method for extracting phenolic acids from propolis samples. Higher temperatures in the extraction process result in thermal degradation and the initiation of oxidation reactions [7]. Namely, benzoic acid derivatives like gallic acid can undergo decarboxylation at elevated temperatures. In addition, heating can accelerate oxidation reactions, which can result in the formation of quinones and other oxidation products. Lastly, at high temperatures, phenols may participate in Maillard reactions. Therefore, our results are in line with the literature indicating that the heating extraction procedure has a negative effect on the stability of benzoic acid derivatives and that solvent extraction enhanced with ultrasound is a more suitable method. UAE minimizes this risk by operating at lower temperatures, thereby maintaining the integrity of the phenolic compounds. In addition to the foregoing, it has been demonstrated that temperatures above 60 degrees Celsius have a significant effect on propolis oxidation, and when selecting the preferred extraction procedure, the matrix composition, which in this case is very diverse and complex, should be considered [18].

At the beginning of the manuscript, it was demonstrated how increasing the duration of ultrasound affects the increased decomposition of phenolic components in the methanol solution. This implies the fact that phenols are labile components, and that ultrasound is a very invasive method of extraction. The goal of the study was to examine the behavior of the same phenolic components, but when they were added to a solution of a complex matrix. For this purpose, we chose propolis, as it is a complex matrix with very lipophilic properties. In the experiment, the stability of propolis extracts was investigated at various ultrasound durations and solvent pH levels. The results showed that by increasing the duration of ultrasound, the recovery of added phenolic components (especially flavonoids) mostly improved, implying strong connections between phenols and the propolis matrix. Changes in pH influenced phenol recovery, with neutral pH favoring flavonoids and low pH favoring phenolic acids. However, these general rules do not apply to all analyzed phenolic compounds, and to ensure successful extraction, the procedure should be calibrated to one or a group of phenolic components with similar physicochemical properties. Because USE is an invasive method, each phenolic component requires a specific duration of ultrasound to extract it from the complex lipophilic matrix of propolis while also preventing decomposition. HRE was used as a standard control method and compared to USE, it proved to be a method that negatively affects the amount of extracted phenols from propolis.

## 4. Conclusions

This study demonstrates that ultrasound-assisted extraction (USE) outperforms the traditional heating reflux extraction (HRE) method for isolating phenolic compounds in propolis. The best extraction solvent was a 50/50 (*v*/*v*) mixture of methanol and water. This combination has a balanced polarity and is effective at solubilizing both polar phenolic acids and less polar flavonoids. USE significantly improved the recovery of flavonoids and phenolic acids, with higher recovery for all analyzed polyphenols: naringenin, quercetin, luteolin, rhamnetin, hesperetin, and quercitrin, and phenolic acids: *p*HBA, gallic acid, 3,4-DHBA, vanillic acid, and cholorogenic acid. Naringenin, quercetin, rhamnetin, and hesperetin showed better recovery with higher USE time (15 min), whereas almost all analyzed phenolic acids showed 100% recovery in every analyzed condition (except for vanillic and chlorogenic acid). However, according to the findings, a 10 min ultrasound duration is ideal for breaking down the propolis matrix without causing excessive degradation of the phenolic compounds. This duration strikes an appropriate balance between extraction efficiency and compound stability. USE operates at lower temperatures, preserving the stability and bioactivity of these sensitive compounds, which often degrade under the high temperatures of HRE. The study underscores the necessity of optimizing extraction parameters, such as solvent composition, pH, and ultrasonic duration, to achieve the highest recovery and stability of phenolic compounds.

Still, it was impossible to choose the single optimal extraction condition that would produce the highest recovery and highest yield of all phenolic compounds. It is critical to investigate the precise conditions that reduce degradation while increasing yield for each polyphenol. Furthermore, investigating the effects of ultrasonic frequency and power on the extraction efficiency of specific phenolic compounds may result in more refined and effective protocols. Overall, tailoring the USE to each polyphenol in propolis will significantly improve the quality and efficacy of the extracts.

## Figures and Tables

**Table 1:** Phenolic standard optimization parameters for LC/MS/MS analysis in negative ionization mode.

Standard	RT	Q1 *m*/*z*	Q3 *m*/*z*
gallic acid	2.98	169	125
3,4-DHBA	4.62	159	108.8
chlorogenic acid	7.46	353	191
*p*HBA	7.78	137	93
vanillic acid	9.42	167	152
*p*-coumaric acid	13.39	163	119
quercitrin	17.30	447	300, 271.2
luteolin	23.46	285	133
quercetin	23.48	301	151
naringenin	27.47	271	151, 118.9
apigenin	27.75	269	117
kaempferol	29.14	285	151
hesperetin	29.40	300.9	164.1
rhamnetin	31.20	315	165
pinocembrin	31.96	255	212, 145

**Table 2:** LogD and logS values of analyzed phenolic compounds [13].

	Phenolic Compound	logD		logS	
		pH = 2	pH = 7	pH = 2	pH = 7
**flavonoids**	naringenin	2.84	2.78	−2.73	−2.67
	quercetin	2.16	1.41	−2.49	−1.73
	apigenin	2.71	2.13	−3.31	−2.72
	kaempferol	2.46	1.71	−0.44	−2.14
	luteolin	2.4	1.82	−2.9	−2.31
	rhamnetin	2.3	2.06	−2.9	−2.65
	hesperetin	2.68	2.62	−2.73	−2.67
	quercitrin	0.9	0.15	−3.05	−2.29
	pinocembrin	3.14	3.08	−3.14	−3.08
**phenolic acids**	*p*HBA	1.32	−1.24	−1.11	0.88
	gallic acid	0.71	−2.22	−0.32	1.66
	3,4-DHBA	1.02	−1.74	−0.72	1.27
	vanillic acid	1.16	−1.59	−1.15	0.84
	chlorogenic acid	−0.32	−3.56	−1.85	0.10

**Table 3:** Concentration of the analyzed flavonoids in propolis extracted by ultrasound-assisted extraction (USE) under different extraction parameters (extraction time and pH) and heat reflux extraction (HRE).

Extraction Modes		Conc./(mg/g)								
USE Time/min	pH	Naringenin	Quercetin	Apigenin	Kaempferol	Luteolin	Rhamnetin	Hesperetin	Quercitrin	Pinocembrin
5	2	0.4680 ± 0.1002	0.6014 ± 0.0520	1.3334 ± 0.0886	1.3418 ± 0.1088	0.1928 ± 0.0012	0.2394 ± 0.0016	0.0500 ± 0.0030	0.0000 ± 0.0000	3.2838 ± 1.2248
	7	0.4272 ± 0.0302	0.8866 ± 0.1418	1.3404 ± 0.1344	1.8248 ± 0.3544	0.2112 ± 0.0384	0.4414 ± 0.1220	0.0652 ± 0.0048	0.0000 ± 0.0000	3.6332 ± 1.2276
10	2	0.3076 ± 0.0036	0.3474 ± 0.1200	0.9094 ± 0.0320	1.7328 ± 0.0356	0.0128 ± 0.0350	0.1596 ± 0.0676	0.0324 ± 0.0024	0.0000 ± 0.0000	1.7784 ± 0.2494
	7	0.4022 ± 0.0672	0.6058 ± 0.1062	1.3896 ± 0.3340	1.5982 ± 0.0357	0.1832 ± 0.0048	0.2666 ± 0.0144	0.0324 ± 0.0040	0.0000 ± 0.0000	2.4356 ± 0.5466
15	2	0.3690 ± 0.1240	0.4371 ± 0.0240	1.3560 ± 0.0624	1.1164 ± 0.0344	0.1908 ± 0.0244	0.1988 ± 0.0072	0.0344 ± 0.0046	0.0000 ± 0.0000	2.1800 ± 0.4680
	7	0.3678 ± 0.0328	0.5008 ± 0.1026	1.0826 ± 0.0612	1.1396 ± 0.0468	0.1896 ± 0.2114	0.2274 ± 0.0080	0.0382 ± 0.0054	0.0000 ± 0.0000	2.2144 ± 0.2554
HRE		0.6484 ± 0.1166	0.9066 ± 0.1762	1.2832 ± 0.0680	2.3370 ± 0.3886	1.2832 ± 0.0682	0.3752 ± 0.0782	0.0590 ± 0.0088	0.0008 ± 0.0002	0.9604 ± 0.1002

**Table 4:** Concentration of the analyzed phenolic acids in propolis extracted by ultrasound-assisted extraction (USE) under different extraction parameters (extraction time and pH) and heat reflux extraction (HRE).

Extraction Modes		Conc./(mg/g)					
USE Time/min	pH	*p*HBA	Gallic Acid	3,4-DHBA	Vanillic Acid	*p*-Coumaric Acid	Chlorogenic Acid
5	2	0.1980 ± 0.0028	0.0000 ± 0.0000	0.0803 ± 0.0073	0.1171 ± 0.0286	4.9544 ± 0.5600	0.0000 ± 0.0000
	7	0.0923 ± 0.0060	0.0000 ± 0.0000	0.0499 ± 0.0115	0.0197 ± 0.0026	3.7045 ± 0.1493	0.0145 ± 0.0002
10	2	0.0698 ± 0.0053	0.0000 ± 0.0000	0.0521 ± 0.0004	0.0515 ± 0.0112	2.7859 ± 0.1140	0.0117 ± 0.0002
	7	0.2773 ± 0.0130	0.0000 ± 0.0000	0.0660 ± 0.0090	0.0917 ± 0.0013	8.0368 ± 0.4338	0.0000 ± 0.0000
15	2	0.1399 ± 0.0060	0.0019 ± 0.0000	0.0477 ± 0.0010	0.0644 ± 0.0012	6.3777 ± 0.5231	0.0147 ± 0.0000
	7	0.1169 ± 0.0029	0.0000 ± 0.0000	0.0518 ± 0.0060	0.0488 ± 0.0199	4.1144 ± 0.0108	0.0000 ± 0.0000
HRE		0.1634 ± 0.0124	0.0006 ± 0.0008	0.0705 ± 0.0028	0.0539 ± 0.0159	6.9122 ± 0.4006	0.0093 ± 0.0008

## Data Availability

The original contributions presented in the study are included in the article/supplementary material, further inquiries can be directed to the corresponding author.

## Supplementary Materials

The following supporting information can be downloaded at: https://www.mdpi.com/article/10.3390/foods13132020/s1, Figure S1: The recovery of apigenin, kaempferol and pinocembrin after USE extraction (5, 10 and 15 min) with methanol/water solution at different pH (2 and 7); Figure S2: The recovery of apigenin, kaempferol and pinocembrin after HRE; Figure S3: The recovery of *p*-coumaric acid after USE extraction (5, 10 and 15 min) with methanol/water (50/50 *v*/*v*) solution at different pH (2 and 7); Figure S4: The recovery of *p*-coumaric acid after HRE.
