# Supplementary material for: Stability of Propolis Phenolics during Ultrasound-Assisted Extraction Procedures
Source: Foods. 2024 Jun 26;13(13):2020. doi: 10.3390/foods13132020 (PMC11240959; doi:10.3390/foods13132020)
Supplement: Supplementary file 1 [file foods-13-02020-s001.zip › foods-3050105-supplementary.pdf]

Supplementary files

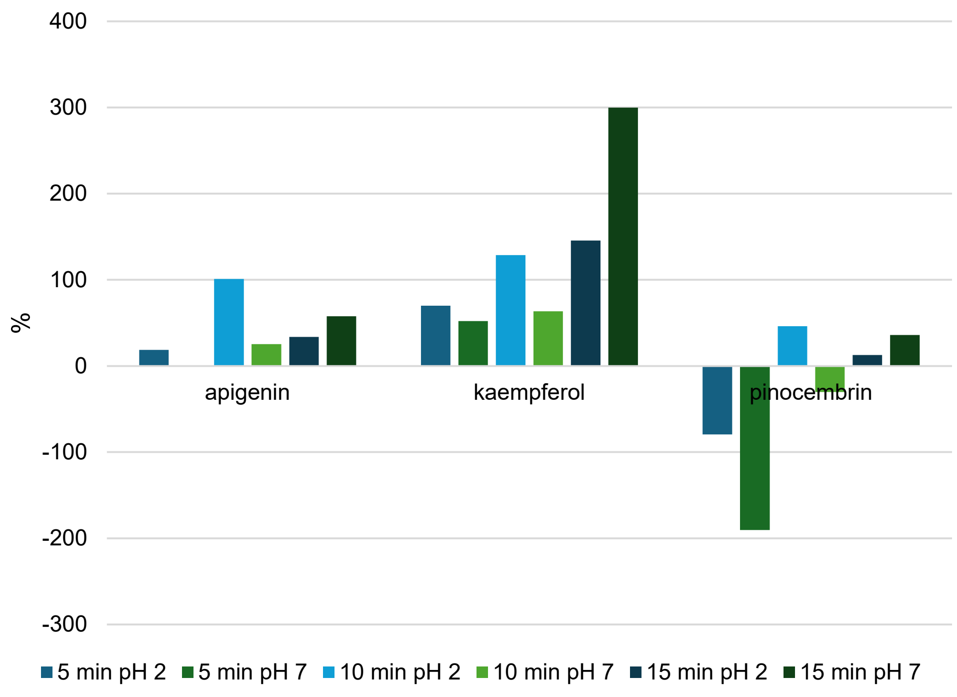

Fig. S1 The recovery of apigenin, kaempferol and pinocembrin after USE extraction (5, 10 and 15 min) with methanol/water (50/50 v/v) solution at different pH (2 and 7).

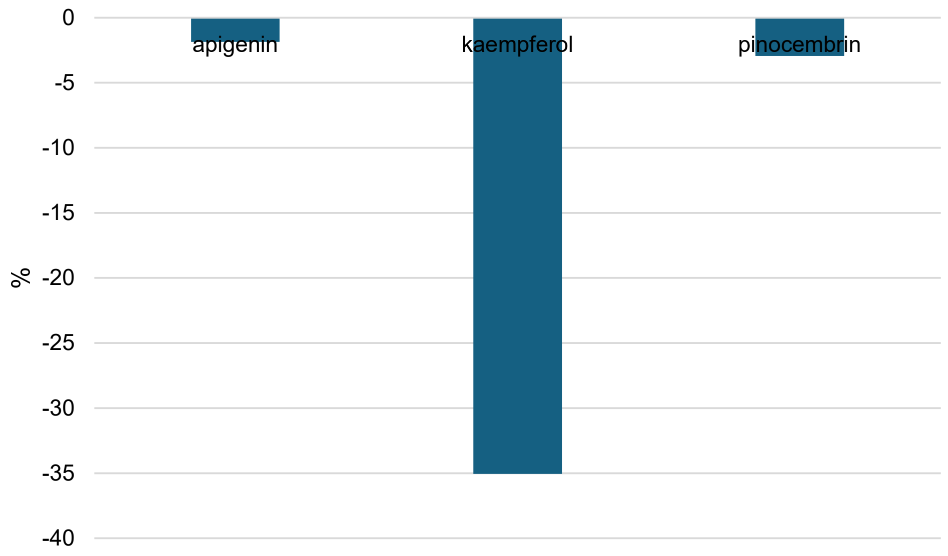

Fig. S2 The recovery of apigenin, kaempferol and pinocembrin after HRE.

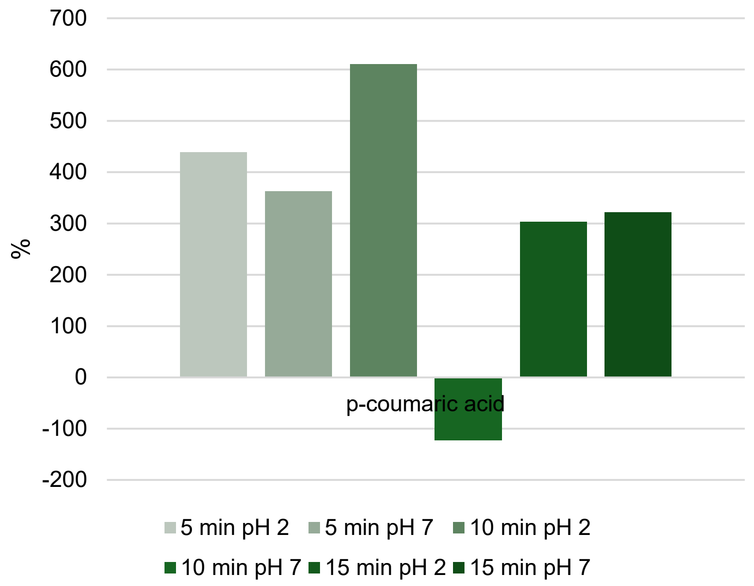

Fig. **S3** The recovery of *p*-coumaric acid after USE extraction (5, 10 and 15 min) with methanol/water (50/50 v/v) solution at different pH (2 and 7).

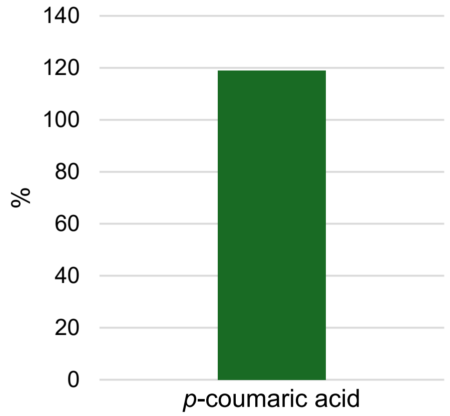

Fig. S4 The recovery of *p*-coumaric acid after HRE.
